# Male rhesus macaques use vocalizations to distinguish female maternal, but not paternal, kin from non-kin

**DOI:** 10.1007/s00265-015-1979-9

**Published:** 2015-07-28

**Authors:** Dana Pfefferle, Angelina V. Ruiz-Lambides, Anja Widdig

**Affiliations:** Junior Research Group of Primate Kin Selection, Department of Primatology, Max-Planck Institute for Evolutionary Anthropology, Leipzig, Germany; Behavioural Ecology Research Group, Institute of Biology, University of Leipzig, Leipzig, Germany; Cognitive Neuroscience Laboratory, German Primate Center, Göttingen, Germany; Leibniz-Science Campus Primate Cognition, German Primate Center and Georg-August-University, Göttingen, Germany; Caribbean Primate Research Center-Cayo Santiago, University of Puerto Rico, Puerto Rico, USA

**Keywords:** Male kin recognition, Dispersing sex, Vocalization, Playback experiment, Maternal vs paternal kin, Degree of relatedness

## Abstract

Recognizing close kin and adjusting one’s behavior accordingly (i.e., favor kin in social interactions, but avoid mating with them) would be an important skill that can increase an animals’ inclusive fitness. Previous studies showed that philopatric female rhesus macaques (*Macaca mulatta*) bias their social behavior toward maternal and paternal kin. Benefits gained from selecting kin should, however, not only apply to the philopatric sex, for which the enduring spatial proximity facilitates kin discrimination. Given that dispersal is costly, the dispersing sex may benefit from migrating together with their kin or into groups containing kin. In male rhesus macaques, natal migrants bias their spatial proximity toward familiar male kin rather than familiar non-kin. Here, we set up playback experiments to test if males use the acoustic modality to discriminate familiar female kin from non-kin in a non-sexual context. Males responded differently to the presentation of “coo” calls of related and unrelated females, with their reaction depending on the interaction between kin-line (maternal vs paternal kin) and degree of relatedness (*r* = 0.5, 0.25). Specifically, males were more likely to respond to close kin compared to more distant kin or unrelated females, with this effect being significant in the maternal, but not paternal kin-line. The present study adds to our knowledge of kin recognition abilities of the dispersing sex, suggesting that male rhesus macaques are also able to identify kin using the acoustic modality. We discuss that the probability of response might be affected by the potential benefit of the social partner.

## Introduction

Gregarious animals form differentiated social relationships with other group members (Hinde 1976). While most of such relationships are weak, some are very strong, enduring, and are characterized by frequent affiliation, close spatial proximity, tolerance, and agonistic aiding (Silk et al. 2010). One of the major factors influencing social bonding is kinship (Gouzoules and Gouzoules 1987), with its reasoning being rooted in the acquisition of inclusive fitness (Hamilton 1964) via both preferential treatment of close relatives in pro-social interactions (“nepotism”, Sherman 1980) and balancing the benefits of mating with close kin against the costs of inbreeding depression (“optimal outbreeding”, Szulkin et al. 2013). To what extent an individual can enhance its fitness depends on its ability to identify and subsequently select relatives in social interactions (“kin selection”, Maynard Smith 1964).

As there is discontinuity in the use of the terms “kin recognition”, “kin bias”, and “kin discrimination”, we defined our use of these terms in accordance to Penn and Frommen (2010). We used the term kin recognition to describe the animals’ ability to identify, distinguish, and classify kin vs non-kin. The term kin bias is used for any differential treatment of kin and non-kin, and kin discrimination refers to the ability to distinguish (i.e., kin recognition) and respond differently (i.e., kin bias) toward kin and non-kin. It is worth noting that kin recognition does not necessarily result in kin bias (Mateo 2002), and kin bias does not require kin recognition (Penn and Frommen 2010). The mechanism(s) underlying kin recognition and kin bias can vary among species and may reflect the social environment (Gerlach and Lysiak 2006). Potential mechanisms by which kin are discriminated are categorized into four classes: spatial distribution, familiarity, phenotype matching, and “direct genetic detection” (*sensu* “recognition alleles” or the “green beard effect”). For reviews on the underlying mechanisms, see, e.g., Tang-Martinez (2001) and Rendall (2004).

Kin recognition and kin bias are taxonomically widespread phenomena, being reported from single cell organisms to primates (e.g., Fletcher and Michener 1987; Queller et al. 2003). Preference tests in insects (e.g., cockroach, *Blattella germanica*: Lihoreau et al. 2007), fish (e.g., sticklebacks, *Gasterosteus aculeatus*: Frommen et al. 2007; zebrafish, *Danio rerio*: Gerlach and Lysiak 2006), and anuran amphibians (for a review, see Blaustein and Waldman 1992) support phenotype matching (i.e., the use of either self or a known kin as a template to assess kinship) as underlying mechanisms in kin discrimination. The cooperative aggregation of social amebas (Queller et al. 2003) is generally referred to as one of the rare examples for direct genetic detection. Mammals are special in the way that the disproportional investment of parents in their offspring produces a social framework in which maternal kinship and familiarity are particularly closely associated. Consequently, a female’s offspring are familiar with other maternal kin (such as aunts, cousins), with familiarity being mediated among maternal siblings through their common mother. Familiarity is therefore the most likely mechanism of maternal kin discrimination in mammals (e.g., white-footed mouse, *Peromysus leucopus*: Tang Halpin and Hoffman 1987; European polecat, *Mustela putorius*: Lode 2008; reviewed for non-human primates: Chapais 2001). Nevertheless, evidence for phenotype matching is also prevalent in mammals (e.g., golden hamster, *Mesocricetus auratus*: Mateo and Johnston 2000; beaver, *Castor candensis*: Sun and Mueller-Schwarze 1997).

Regardless of the possible underlying mechanism(s), most research on mammals has been devoted to *maternal* kin recognition or kin bias, respectively (recognition: e.g., northern fur seal, *Callorhinus ursinus*: Insley 2000; domestic goat, *Capra hircus*: Briefer and McElligott 2011; rhesus macaque, *Macaca mulatta*: Gouzoules 1984; Rendall et al. 1996; Barbary macaque, *Macaca sylvanus*: Hammerschmidt and Fischer 1998; bias: e.g., house mice, *Mus domesticus*: König 1989, spotted hyena, *Crocuta crocuta*: Wahaj et al. 2004; Barbary macaque: Kuester et al. 1994; reviewed for non-human primates: Chapais 2001; Silk 2002). Although paternal kin are less affiliated, hence less familiar, than maternal kin (Widdig et al. 2001, 2002), support for *paternal* kin recognition or kin bias is accumulating too (recognition: e.g., Belding’s ground squirrel, *Spermophilus beldingi*: Mateo 2002; gray mouse lemur, *Microcebus murinus*: Kessler et al. 2012; rhesus macaque: Pfefferle et al. 2014a, b; bias: e.g., spotted hyena: Wahaj et al. 2004; rhesus macaque: Widdig et al. 2001; yellow baboon, *Papio cynocephalus*: Silk et al. 2006; mandrill, *Mandrillus sphinx*: Charpentier et al. 2007).

Generally, research on kin discrimination is heavily biased toward the philopatric sex, for which the number of relatives living together is likely to be higher than for the dispersing sex after departure from the natal group. Even after dispersal, however, animals have the opportunity to increase their inclusive fitness (Langergraber 2012). Hence, despite fewer opportunities for kin bias after dispersal, selection should have favored kin bias in the dispersing sex too. Two of the main contexts in which the emigrating sex can achieve fitness benefits are kin-biased dispersal and the care for/by relatives (for a review, see Langergraber 2012). In kin-biased dispersal, animals either (i) emigrate together with their kin (paternal and/or maternal kin) or (ii) immigrate into groups that contain previously immigrated kin (e.g., maternal-, or peternal-siblings). To date, only a few studies have investigated kin bias in the dispersing sex (e.g., Albers and Widdig 2013; Widdig et al. 2015), which is probably due to the difficulty of getting comprehensive genetic data for determining relatedness among individuals in different groups.

Which of the two sexes in a species disperses depends, among other things, on socioecological factors such as feeding competition, social tolerance, and risk of predation (Schülke and Ostner 2012). Cercopithecines are characterized by female philopatry and male dispersal (Schülke and Ostner 2012). Within this primate subfamily, males of some species were found to preferentially disperse into groups where their maternal brothers live, spending more time together as well as support each other (e.g., vervet monkey, *Cercopithecus aethiops*: Cheney and Seyfarth 1983; rhesus macaque: Meikle and Vessey 1981). In such a case, the presence of brothers was associated with a longer tenure in the non-natal group (Meikle and Vessey 1981). A more recent study on natal dispersal in rhesus macaques suggests that both maternal and paternal relatedness influence the spatial proximity among familiar male migrants (i.e., born and grown up in the same group) that either emigrated together into a new group or moved at different times into the same non-natal group (Albers and Widdig 2013). Spatial proximity was also influenced by the degree of relatedness, with more closely related kin sitting closer to each other than more distantly related kin or unrelated individuals (Albers and Widdig 2013).

In addition to discriminating same-sex kin, the discrimination of opposite-sex kin may also lead to fitness benefits, e.g., through optimal outbreeding (Szulkin et al. 2013) and directing mating effort (“care-then-mate,” Ménard et al. 2001). Also, outside the mating context, the preferential association with opposite-sex kin can increase an individual’s fitness. The close association between son and mother, for instance, has been shown to result in a male’s advancement in rank in his natal group (if mother is of high rank, Chapais 1983). Furthermore, in species with male dispersal, related and unrelated females may potentially promote a male’s integration process into a new group and hence decrease dispersal costs (Hill 1990; Gould 1996).

Recognizing kin requires cues reflecting genetic relatedness or cues encoding individual identity that are linked to relatedness. What type of cue is used is likely to depend upon the key sensory modalities of a species as well as on the behavioral context (Widdig et al. 2002). While the appearance of a given individual can be used to identify kin in situations of close visual contact, the ability to vocally identify kin would be an important adaption facilitating social communication from a larger distance or with constrained visual contact. Vocalizations convey information about the vocalizer, such as its identity (Semple 2001; Rendall 2003; Price et al. 2009), sex (Ey et al. 2007), age (Hammerschmidt et al. 2000), size (Pfefferle and Fischer 2006), hormonal state (Pfefferle et al. 2008; Pfefferle et al. 2011), and very likely kinship (Gouzoules and Gouzoules 1990; Hauser 1991; Pfefferle et al. 2014b). The majority of studies examining the salience of vocalizations in the context of kin selection investigated the recognition among mothers and their offspring (e.g., Gouzoules 1984; Hammerschmidt and Fischer 1998) or other maternally related, familiar females (Rendall et al. 1996). In rhesus macaques, the presentation of coo calls, a vocalization uttered in prosocial contexts, elicited a stronger response in close maternal kin compared to more distant maternal kin or maternally unrelated females (Rendall et al. 1996). Recently, similar results were found for females responding differently toward calls of their paternal half-sisters and unrelated females (Pfefferle et al. 2014b). As this finding occurred independent of familiarity between caller and receiver, it suggests phenotype matching with some sort of information about relatedness being encoded in this call type. While these two studies examined the ability of females to vocally recognize female kin, we are aware of only one study that tested the ability to vocally distinguish opposite-sex relatives. In gray mouse lemurs, females are less likely to respond to advertisement calls of their fathers in comparison to unrelated males (Kessler et al. 2012). Surprisingly, to date, no study tested whether males too recognize their female kin, and if so, which cues are used in this process.

Here, we investigate whether males use the acoustic modality to identify their familiar female kin. We choose rhesus macaques, a species living in multi-male, multi-female groups, with female philopatry, and male dispersal (Drickamer and Vessey 1973). In this species, male reproduction is skewed toward a few males siring multiple offspring with different females, i.e., paternal half-siblings (Widdig et al. 2004; Dubuc et al. 2011; Dubuc et al. 2014). Although paternal kin bias has been reported, strongest social bonds exist among maternal kin (Widdig et al. 2001). Most males leave their natal group around puberty (Berard 1990), undertaking multiple dispersals thereafter (Drickamer and Vessey 1973). We presented males with coo calls of related vs unrelated females that coresided in the same social group. First, we tested whether males use the acoustic modality to distinguish between their female kin and non-kin. Second, we investigated whether the males’ ability to identify kin depends upon the degree of relatedness (i.e., parent-offspring: *r* = 0.5, half-siblings of the opposite sex: *r* = 0.25) and/or differs between kin-lines (i.e., maternal and paternal kin-line). Based on previous results on female rhesus macaques (Rendall et al. 1996; Pfefferle et al. 2014b), we predicted that males use acoustic cues to distinguish kin from non-kin and respond stronger or more often to calls of more closely related individuals. Due to the importance of maternal ties in this species (Widdig et al. 2001), we expected this effect to be most pronounced in the maternal kin-line, but predicted paternal kin to also elicit a stronger response than unrelated individuals. In order to avoid confounding motivation based on a potential preference for unrelated callers in the mating context vs a potential preference for kin callers in a non-sexual context, we performed playback experiments outside the mating season. Playbacks were performed in multiple social groups of a large free-ranging population for which genetic relatedness was determined from an extensive pedigree constructed using molecular markers.

## Methods

### Study site and subjects

We conducted the study on the free-ranging rhesus macaque population on Cayo Santiago (18° 09′ N, 65° 44′ W), Puerto Rico (details in Rawlins and Kessler 1986). During the study, the island was inhabited by approximately 1200 individuals, belonging to 6 naturally formed social groups. From the demographic data collected by the Caribbean Primate Research Centre (CPRC), information on maternal kinship, date of birth, natal group, and duration of group membership was available for each individual of the study population. Rhesus macaques on Cayo Santiago are habituated to human observers and used to experimental equipment. Subjects were individually recognized using tattoos, ear notches, and natural markings. In total, we tested 52 sexually mature males (mean age 8.24 years, range 3.5–21.7 years) from all groups. Test subjects and callers were familiar with one another, defined as being coresident in the same social group (see below). To exclude a mating context, the study was performed during the birth seasons (Jul–Dec 2011 and Sept–Dec 2012).

### Vocal recordings

To conduct playback experiments, we recorded coo calls of adult females of known relatedness to our male test subjects (see below). Coo calls are harmonically rich vocalizations encoding information about an individuals’ identity (Rendall et al. 1996, 1998), and likely relatedness (Pfefferle et al. 2014b). They are produced in different contexts, such as food acquisition and group progression, with the acoustic structure of these “coo types” being distinctive (e.g., Rowell and Hinde 1962; Hauser 1991). For presentation, we only used calls free of background noise, uttered during the same context (i.e., group progression), and that were recorded in the same or the previous year of the experiment. Selected calls were standardized to the same peak sound pressure level of 68 dB measured at 5-m distance from the loudspeaker (Sound Level Meter: PeakTech 5055, Ahrensburg, Germany). For more information on call recording, processing, and stimuli preparation, see Pfefferle et al. (2014b).

### Female dominance hierarchy

In rhesus macaques, females form a stable linear hierarchy that is organized along matrilines of closely ranked and maternally related individuals. We collected hierarchy information of adult females resident in all six groups living on Cayo Santiago during our study period. Hierarchy was based on the direction and outcome of agonistic interactions, recorded ad libitum (Altmann 1974).

### Playback experiment

We ran four playback conditions to test the males’ ability to distinguish between calls of females with different coefficients of relatedness to them (*r* = 0.5, *r* = 0.25, *r* ≤ 0.063, see below “Determination of kinship”) and from different kin-lines (maternal, paternal, see below “Determination of kinship”). Each male was tested twice: once with a call of a related female and once with a call of an unrelated female. The order of presentations was counterbalanced and separated by at least 1 day. We tested a total of 52 males in the following four conditions: (i) “son-mother” condition—males were presented with a call of their mother (*r* = 0.5) vs an unrelated (*r* ≤ 0.063) female (*N* = 28 playbacks, 14 kin and 14 non-kin); (ii) “maternal half-sibling” condition—males were presented with a call of their maternal half-sister (*r* = 0.25) vs an unrelated female (*N* = 28 playbacks, 14 kin and 14 non-kin); (iii) “father-daughter” condition—males were presented with a call of their daughter (*r* = 0.5) vs an unrelated female (*N* = 24 playbacks, 12 kin and 12 non-kin); (iv) “paternal half-sibling” condition—males were presented with a call of their paternal half-sister (*r* = 0.25) vs an unrelated female (*N* = 24 playbacks, 12 kin and 12 non-kin). Note, while in the first two conditions we tested kin recognition within the maternal kin-line, the two latter conditions tested the paternal kin-line. We kept the age disparity between the related and unrelated female presented in playbacks at a minimum (mean ± SD 1.3 ± 1.1 years). Additionally, we minimized rank disparities between the callers using hierarchy information (see “Female dominance hierarchy”), choosing the non-kin call donor with the minimum rank distance to the kin call donor (Pfefferle et al. 2014b). Furthermore, we controlled for group membership, with males and their female call donors being coresident for at least 1.5 years (mean ± SD 5.7 ± 2.6 years) prior to the study. Callers served on average 1.7 times as call donor in different tests. If callers were used repeatedly, different calls of that individual were presented. Each test male participated in only one of the four conditions.

As female *maternal* kin are only available for males in their natal group, tests of the son-mother and maternal half-sibling condition were performed with males being resident in their natal group. Female *paternal* kin, on the other hand, may be found both in a male’s natal or non-natal group. Based on availability of paternal siblings and the match of the kin and non-kin call donors in age and rank, we conducted tests of the “paternal half-sibling” condition inside (*N* = 9) and outside (*N* = 3) the males’ natal group. There was no indication that the three non-natal males showed a different looking pattern in response to call donors than males tested in their natal group. As males typically fathered offspring outside their natal group, 11 out of 12 males were tested outside their natal group in the “father-daughter” condition. In total, 52 males and 72 females (kin and non-kin call donors) participated in this study.

Note that this study was not designed to test for the underlying mechanisms of kin recognition. This would involve testing unfamiliar individuals (see Pfefferle et al. 2014b) and constitutes a step which has to follow after verifying that males use vocalizations for kin recognition (this study). Furthermore, we acknowledge that it would have been interesting to test the ability of males to acoustically distinguish between male maternal-, paternal-, and non-kin. This was, however, not possible, because males emitted fewer coo calls than females (Pfefferle et al. unpublished data) providing us with an insufficient number of male playback stimuli.

### Assigning parentage

To determine parenthood, we used a long-term genetic database for this population, encompassing 3735 individuals (for details, see Pfefferle et al. 2014b). Of the 124 individuals used in this study, all mothers (*N* = 96 unique dams) were genetically determined and used in subsequent paternity analyses. For paternity assignments, all males in the population older than 1250 days (based on earliest age at reproduction, Bercovitch et al. 2003) and present on the island at least 200 days prior to the birth of a given individual (based on mean days ± SD gestation length: 166.5 ± 7.4, Silk et al. 1993) were considered as potential sires. Mother-sire-offspring trios were genotyped on 28 or 29 (mean = 28.95, SD ±0.09) common loci. Paternity was assigned using a combination of exclusion and likelihood methods. In all but one cases, the assigned sire had no mismatch with the mother-offspring pair, and all other candidate sires had been excluded by at least two loci (*N* = 94 unique sires). In the remaining case, all potential sires sampled had been excluded by at least two mismatches when compared with the mother-offspring pair, suggesting that the actual sire was not sampled. However, we were able to include this case, because the individual was used as a mother in a son-mother condition not requiring the ID of her maternal grandfather for kinship verification.

For maternal and paternal half-siblings and all non-kin relationships, we further aimed to assign the grandparents. For the 86 unique dams (96 unique dams minus 10 mothers from the son-mother condition for which known grandparents are not required), we were able to genetically confirm 61 unique mothers (70.93 %), i.e., the grandmothers of the target individual. In the remaining cases, no genetic sample of the demographic grandmother was available. However, due to the low level of demographically misassigned mothers in the entire study population (*N* = 80 out of 3247 genetically confirmed mothers, 2.46 %), we felt confident to use the demographically assigned grandmothers in those cases. A total of 53 (61.63 %) unique maternal grandfathers could be genetically determined by excluding all other potential grandfathers at two or more loci. In the remaining cases, all candidates could be excluded by at least two mismatches indicating that the actual grandfather was not sampled.

Based on the 83 unique sires (94 sires minus 11 sires from the father-daughter condition for which known grandparents are not required), we genetically identified 65 (78.31 %) paternal grandmothers and 62 (74.70 %) paternal grandfathers, excluding all other candidates by at least two mismatches. In the remaining cases, no genetic sample was available. Given the high agreement between demographically assigned and genetically confirmed mothers (see above), we used the demographically assigned match as paternal grandmother. In cases of missing grandfather information, we ensured relatedness or non-relatedness via additional exclusion criteria (see below).

All relatedness data between individuals were additionally confirmed at the 95 % confidence level by the maximum likelihood method calculated by CERVUS 3.0 (Kalinowski et al. 2007).

### Determination of kinship

Pedigrees up to the grandparent generation were used to generate triads consisting of a test male, an unrelated female, and a related female—according to the test condition, either a mother, a daughter, a maternal or paternal half-sister. We defined dyads that shared relatedness through the mother as maternal kin-line (son-mother and maternal half-sibling condition) and dyads sharing relatedness through the father as paternal kin-line (father-daughter and paternal half-sibling condition). Half-siblings either shared the same mother (maternal half-siblings) or father (paternal half-siblings), but differed in the identity of the respective other parent and grandparents. Unrelated females were defined as individuals that have no ancestor in common with the test male, up to the grandparent generation. In cases where the maternal or paternal grandfather of an individual was unknown, we used two exclusion criteria to ensure that the female was indeed unrelated to our test male: (i) the putative grandfather of individual A could be excluded as the grandfather of individual B by at least 2 mismatches or (ii) the grandfather of individual A was too young (<1250 days at time of conception) to have sired either the father or mother of individual B.

### Playback procedure and analysis

A detailed description of how we conducted and analyzed the playback experiments can be viewed in Pfefferle et al. (2014b). Briefly, playbacks were broadcasted toward test males from an average distance of 11.7 ± 1.55 m (mean ± SD) and between 0° and ±45° behind his back (0° refers to the position straight behind the male’s back) using a DAVIDactive 5001 loudspeaker (Visonik, Berlin, Germany) concealed behind dense vegetation. The male’s response to the call presentation was recorded with a digital video camera (JVC HD Everio Memory Camcorder, GZ-HM650, Japan). To prevent habituation to the experiment, we aimed at conducting no more than two trials per day and group (mean ± SD 1.35 ± 0.5). Trials were conducted only if the female whose call was going to be presented was out of sight of the test male, if the test male was settled and not engaged in potentially distracting activity (e.g., feeding, grooming others), and if no disturbing noise could be heard.

Video recordings were transferred to iMovie (version: 9.0.4, Apple Inc., USA) and analyzed frame by frame (25 frames/s). The males’ reaction to a playback was classified as a valid response if he turned his head over his shoulder and within an angle of at least ±90° of the loudspeaker. To make the analysis comparable to our previous study (Pfefferle et al. 2014b), the movement of the head had to occur within the first 10 s after the playback call was broadcasted. We chose the 10 s in the first place, because during this time span, interferences from outside, such as loud noise to which the test subject potentially reacts, are limited. If these conditions of timed head movement were not fulfilled, we noted that no response was given. We included moving toward the loudspeaker as response if it occurred within the 10 s after playback start, which happened only twice in 104 trials.

Because males showed a response in only 44 playbacks (42.3 % of all 104 playbacks), a number effectively too low with regard to the complexity of the statistical model (see below), we refrained from measuring the latency and duration of response (see also Pfefferle et al. 2014b) and restricted our analyses to whether or not males responded to the playback call.

### Statistical analysis

To investigate whether the probability that males responded toward a call of a kin vs a non-kin depends on the level of relatedness (*r* = 0.5, *r* = 0.25, *r* ≤ 0.063), the kin-line (maternal vs paternally related) or its interaction, we ran a generalized linear mixed model (GLMM, Baayen 2008) with binomial error structure and logit link-function. In addition, we included the playback order (i.e., whether the kin or the non-kin call was presented first) as fixed effect and the ID of the test subject and caller as random effects. The model was fitted in R v. 2.14.1 (R Core Team 2013) using the function “lmer” of the R-package lme4 (Bates et al. 2011).

We first established overall significance of the full model (Forstmeier and Schielzeth 2011) as compared with a null model (that excluded the main predictors and thus comprised only the intercept, the fixed effect of order of playback presentation, and the random effects), using a likelihood ratio test (LRT, Dobson 2002). We also checked for model stability by excluding data points one by one from the data set and comparing the estimates derived with those obtained from the full model. No indication for influential cases was found. Having demonstrated significance of the full model, we checked for significance of the interaction term by comparing the fit of the full model with that of the reduced version (excluding the interaction term) using an LRT. The interaction term was found to be significant and was therefore kept in the final model. Subsequently, we controlled for this interaction in a post hoc analysis repeating the GLMM (see above), but this time testing the effect of the degree of relatedness separately within the maternal and paternal kin-line.

## Results

Across all playback conditions, 59.62 % of the tested males showed a response (*N* = 30 looked, 1 stood up and walked toward the loudspeaker) when presented with a call of a related female. In contrast, 25 % of the males responded (*N* = 12 looked, 1 stood up and walked toward the loudspeaker) when presented with a call of an unrelated female (see Table 1 for details).

**Table 1:** Summary of results: number of playback trials in which males responded to the presentation of an unrelated (%, *N*)/related (%, *N*) female call, in relation to the maternal and paternal kin-line

*R* value	Maternal	Paternal
≤0.063 vs 0.5	3 (21 %, *N* = 14) / 12 (85.7 %, *N* = 14)	2 (16.6 %, *N* = 12) / 5 (41 %, *N* = 12)
≤0.063 vs 0.25	3 (21 %, *N* = 14) / 9 (64 %, *N* = 14)	5 (41 %, *N* = 12) / 5 (41 %, *N* = 12)

**Fig. 1:**
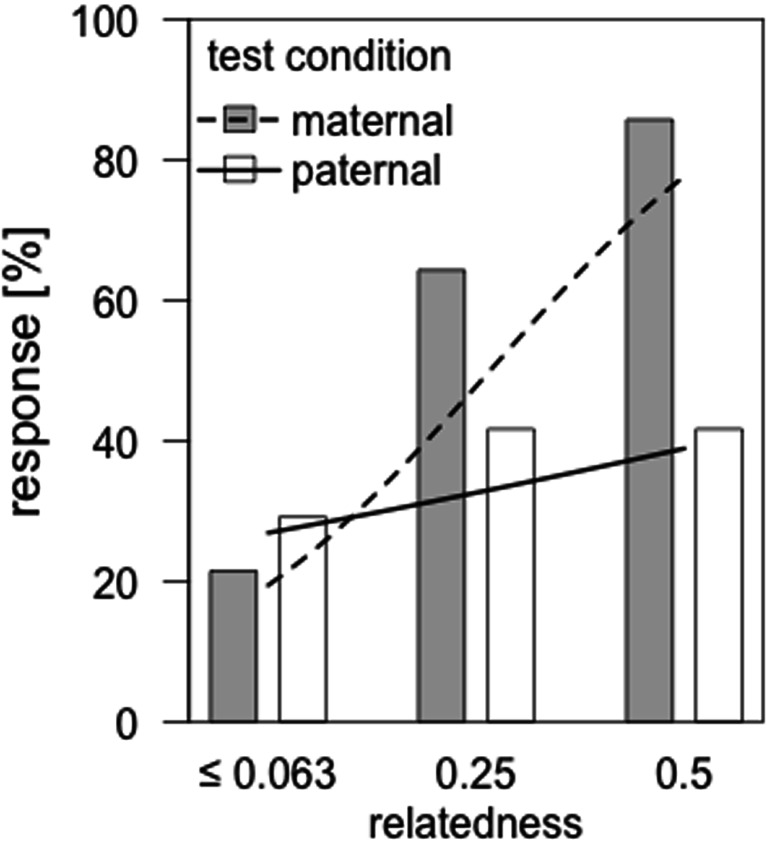
Percentage of males that responded to the presentation of “coo” calls from females with different relatedness to them. In the maternal condition (*gray bars*), we presented unrelated individuals (*r* ≤ 0.063) vs maternal half-sisters (*r* = 0.25) or mothers (*r* = 0.5). Likewise, in the paternal condition (*white bars*), unrelated individuals (*r* ≤ 0.063) vs paternal half-sisters (*r* = 0.25) or daughters (*r* = 0.5) were presented. *Dashed and solid lines* represent the model estimates for male responses toward the different degrees of relatedness within the maternal and paternal kin-line, respectively

A comparison of the full vs the null model revealed that overall, the two predictor variables (degree of relatedness and kin-line) influenced the probability that a male looked toward the broadcasted call (LRT: χ^*2*^ = 21.389, *df* = 1, *p* < 0.001). There was a significant interaction between kin-line and the degree of relatedness (LRT: χ^*2*^ = 5.545, *df* = 1, *p* = 0.019, Table 2, Fig. 1), indicating that the effect of the degree of relatedness differs between kin-lines. Specifically, an increase in the degree of relatedness led to a higher probability of male response within the maternal kin-line (post hoc GLMM: Z = 2.508, *p* = 0.012, Fig. 1), but not the paternal kin-line (post hoc GLMM, Z = 0.770, *p* = 0.427, Fig. 1). In no case did the order in which the calls were presented influence the males’ response (*p* > 0.05).

**Table 2:** Results of the final GLMM testing the effect of relatedness (*r* = 0.5, *r* = 0.25, *r* ≤ 0.063) and kin-line (maternal vs paternal) on the probability of males responding toward the playback

Predictor variable	Estimate	SE	Z	*p*
Intercept	−1.773	0.924	−1.918	0.055
Degree of relatedness				
within maternal line	7.365	2.251	3.272	0.001
within paternal line	1.662	1.684	1.078	0.324
Kin-line (maternal = 0, paternal = 1)	0.387	0.697	0.556	0.578
Playback order (kin presented 1st = 0, 2nd = 1)	0.236	0.486	0.484	0.628
Interaction btw. degree of relatedness and kin-line* (maternal = 0, paternal = 1)	−5.703	2.605	−2.189	0.029

The test subjects ID and the callers ID were included as random effects

*Overall LRT: χ^2^ = 5.545, *p* = 0.0185, *df* = 1

## Discussion

Our study tested the ability of the dispersing sex, here male rhesus macaques, to use the acoustic modality as cue to identify familiar maternal and paternal kin of different degrees of relatedness. We focused on the ability of the dispersing sex to recognize opposite-sex kin in a non-sexual context, as this is a largely neglected aspect among mammalian studies of kin recognition. Overall, the results of our playback experiment indicated that male rhesus macaques distinguished between related and unrelated females using the acoustic modality, with kin-line and degree of relatedness influencing the probability to respond. Specifically, males were more likely to respond to close kin compared to more distant kin or unrelated females, with this effect being significant in the maternal, but not paternal kin-line. Our finding on males recognizing maternal kin is in line with results from previous playback experiments showing that calls of maternally related females elicit a stronger response in females than calls uttered by more distant or unrelated females (Rendall et al. 1996). In contrast to our result on maternal kin recognition, our data on paternal kin recognition does not meet previously found evidence for female rhesus macaques responding differently toward calls of their paternal half-sisters and unrelated females (Pfefferle et al. 2014b).

In rhesus macaques, the rank and reproductive success of a natal male was found to be correlated with the rank of his mother (Smith and Smith 1988). A similar effect of mother’s rank on the reproductive success of her son(s) was reported in bonobos (*Pan paniscus)*, a male philopatric species (Surbeck et al. 2011). This potential reproductive advantage for sons holds as long as they stay in their natal group, which has been suggested to induce sons of high-ranking females to sometimes remain in their natal groups after puberty (Smith and Smith 1988; Dubuc et al. 2011), potentially attain high rank, mate, and even reproduce before finally departing from their natal group. The long association between mothers and their sons might explain why, in our playback study, males responded most often to calls of their mothers. Since mothers maintain close associations with all their offspring, a male becomes familiar with his maternal sisters. As maternal relatedness generally has a strong effect on the formation of social bonds (e.g., rhesus macaques: Kapsalis and Berman 1996; Widdig et al. 2002; savannah baboons, *Papio cynocephalus*: Silk et al. 2006), it is not surprising that males also showed a higher probability to respond to calls of maternal half-sisters in comparison to unrelated females.

A recent developmental study on rhesus macaques, investigating mother-offspring associations from birth to sexual maturation, showed a five times higher probability of maternal aggression toward sons, in relation to daughters, during the first year of offspring life (L. Kulik et al. unpubl. data). This higher level of maternal aggression might reflect the need or opportunity of immature males to also seek social partners outside of their maternal family, such as paternally related males or females, when still living in their natal group. Yet, our study does not provide evidence that males (acoustically) recognize their female paternal kin. One possible explanation for this lack of recognition is that the direct fitness benefits for a male forming an affiliative bond with his female paternal kin might effectively be too low to outweigh the possible costs of nepotism (e.g., injuries resulting from the interventions in conflicts and cooperative defense of food sources, cost due to shared food resources). This imbalance toward the costs might result in a decreased motivation to respond to calls of paternally related females. The low motivation to respond to female calls might as well be rooted in the species dispersal regime. In this respect, connectedness between group members might be less important (hence lower motivation to respond) for the dispersing sex than for the philopatric sex. A non-mutually exclusive explanation for the lack of paternal kin recognition by males in the present study is that males may not as extensively use (or rely on) the acoustic modality as females do. This is supported by the observation that overall males tend to vocalize less frequently than females do (DP et al. unpubl. data). For maternal kin, but not paternal kin or unrelated conspecifics, such potential deficit in vocal use might be compensated by extended familiarity among close maternal kin. However, the lack of experimental support for paternal kin recognition of familiar individuals does not necessarily preclude that males treat paternal kin differently than unrelated individuals. This is because kinship might be inferred by other cues (e.g., olfactory, visual, age proximity), with the transformation between those other cues and the acoustic modality being mediated via animal identity encoded in the acoustic structure (Rendall et al. 1998). In fact, several lines of evidence support that rhesus macaque males bias their behavior toward familiar paternal kin. First, adult male rhesus macaques were found to preferentially affiliate with their offspring independent of the presence of the mother (Langos et al. 2013). Second, after natal dispersal, males were observed to sit closer to their familiar paternal (and maternal) male kin, in comparison to familiar non-kin (Albers and Widdig 2013). Third, males, in contrast to females, bias their interactions towards familiar paternal kin (when compared to non-kin) already prior to their natal dispersal (i.e., at 4 years of age, Widdig et al. 2015), indicating that the time span between birth and natal migration is effectively sufficient to develop behavioral paternal kin bias.

In regard to opposite-sex interactions, most studies on mammals available today focus on kin selection in the context of reproduction. In this respect, mechanisms for inbreeding avoidance between fathers and daughters were reported in, e.g., striped mice (*Rhabdomys pumilio*, Pillay 2002) and mandrills (Charpentier et al. 2005). Considering lower levels of paternal relatedness, e.g., half-siblings, savannah baboons were reported to exhibit less affiliative and sexual behavior among related opposite-sex dyads than among unrelated opposite-sex dyads (Alberts 1999). When screening the literature for paternal kin recognition or kin bias of opposite-sex dyads outside the mating context, studies become sparse. This is because most studies either focus on same-sex dyads (e.g., Kareem and Barnard 1982; Todrank et al. 1998; Widdig et al. 2002; Smith et al. 2003; Silk et al. 2006; Langergraber et al. 2007; Albers and Widdig 2013) or do not distinguish between same- and opposite-sexed kin partners in their analyses (e.g., Mateo 2002; Lehmann et al. 2006; Widdig et al. 2015). Hence, in the latter case, the reported evidence may have been driven by the preferential response toward one sex only. In light of the present study, this might suggest that males bias their behavior toward male, rather than female paternal kin (unfortunately, we were only able to test female, not male paternal kin here). In fact, it would be beneficial for males to disperse with their male paternal kin, which often tend to be of the same age (Widdig et al. 2015). The preference of same-sex kin is supported by a study on rhesus macaques investigating whether adult males and females are able to recognize their own paternal half-siblings based on facial cues alone (Pfefferle et al. 2014a). By controlling for cues indicating individual receptivity, this study provides support for same-sex, but not opposite-sex kin recognition. The lack or decrease in detectability of male-female paternal kin recognition is in line with our current finding of males not preferentially responding to calls of their female paternal kin.

In summary, our study provides the first evidence that male rhesus macaques also use the acoustic modality to identify their maternal kin when other cues are unavailable. Our results show that males did not respond differently to calls of paternal kin vs unrelated females, which might be rooted in the species’ dispersing regime that, apart from the close association among maternal kin, promotes same-sex rather than opposite-sex bonds in a non-sexual context. To verify this, future research should investigate kin recognition and kin bias of both the philopatric and the dispersing sex including same- vs opposite-sex kin dyads.
